# Multi-scale and attention enhanced graph convolution network for skeleton-based violence action recognition

**DOI:** 10.3389/fnbot.2022.1091361

**Published:** 2022-12-15

**Authors:** Huaigang Yang, Ziliang Ren, Huaqiang Yuan, Wenhong Wei, Qieshi Zhang, Zhaolong Zhang

**Affiliations:** ^1^School of Computer Science and Technology, Dongguan University of Technology, Dongguan, China; ^2^Shenzhen Institute of Advanced Technology, Chinese Academy of Sciences, Shenzhen, China; ^3^Rutgers, The State University of New Jersey, New Brunswick, NJ, United States

**Keywords:** violence action recognition, skeleton sequence, multi-scale graph convolution network, attention mechanism, spatiotemporal information

## Abstract

Graph convolution networks (GCNs) have been widely used in the field of skeleton-based human action recognition. However, it is still difficult to improve recognition performance and reduce parameter complexity. In this paper, a novel multi-scale attention spatiotemporal GCN (MSA-STGCN) is proposed for human violence action recognition by learning spatiotemporal features from four different skeleton modality variants. Firstly, the original joint data are preprocessed to obtain joint position, bone vector, joint motion and bone motion datas as inputs of recognition framework. Then, a spatial multi-scale graph convolution network based on the attention mechanism is constructed to obtain the spatial features from joint nodes, while a temporal graph convolution network in the form of hybrid dilation convolution is designed to enlarge the receptive field of the feature map and capture multi-scale context information. Finally, the specific relationship in the different skeleton data is explored by fusing the information of multi-stream related to human joints and bones. To evaluate the performance of the proposed MSA-STGCN, a skeleton violence action dataset: Filtered NTU RGB+D was constructed based on NTU RGB+D120. We conducted experiments on constructed Filtered NTU RGB+D and Kinetics Skeleton 400 datasets to verify the performance of the proposed recognition framework. The proposed method achieves an accuracy of 95.3% on the Filtered NTU RGB+D with the parameters 1.21M, and an accuracy of 36.2% (Top-1) and 58.5% (Top-5) on the Kinetics Skeleton 400, respectively. The experimental results on these two skeleton datasets show that the proposed recognition framework can effectively recognize violence actions without adding parameters.

## 1. Introduction

Recently, individual and group violence in public places has seriously threatened the safety of people's lives and property. With the widespread deployment of video surveillance equipment, video motion understanding and recognition based on computer vision technology has become an effective public security tool for identifying danger and preventing crime. However, the detected targets in surveillance scenes are often affected by background noise, light intensity changes, camera views, and clothing, which requires not only improving the accuracy of the model but also considering the computational cost of the algorithm (Serrano et al., 2018; Ramzan et al., 2019). The existing recognition methods mainly use different modalities as inputs, and learn spatiotemporal features by designing Convolutional Neural Networks (CNN) (Cheng et al., 2021; Ji et al., 2021; Gadelkarim et al., 2022) and Recurrent Neural Networks (RNN) (Liu et al., 2018; Song et al., 2018; Jiang et al., 2020; Shu et al., 2021).

With the development of a graph convolution network (GCN), the skeleton-based approaches have achieved success in violent action recognition due to it can better reduce the model complexity (Senst et al., 2017; Liu Z. et al., 2020; Li M. et al., 2022). The skeleton data is essentially a topological graph, where human joints are represented as vertices and bones are represented as edges of the graph. Although skeleton sequences has comparative advantages over RGB or depth modalities, skeleton based recognition methods still face difficulties and challenges in the following two aspects: (1) In the spatial space, there is spatial information and a certain strong correlation between the neighboring nodes in each frame, and it is necessary to mine the action structure information. (2) In the temporal space, the motion structure of the joint points is important for characterizing the action, which needs to model the long-range temporal information.

As existing work mainly considers a series of convolution operations on a single feature map (Liu Z. et al., 2020; Li M. et al., 2022), which to some extent fails to obtain larger receptive field information. We use the design of a multi-scale approach to obtain larger and more receptive field information, which is beneficial for feature learning of the model and expression. The attention mechanism mainly focuses the model on the main joint points or skeletal edges where certain movements occur, which helps to eliminate redundant dependency information between joint point features, thus effectively capturing the main association information between joint points. Meanwhile, thanks to advanced pose estimation methods (Openpose, Cao et al., 2021) the skeleton information may be extracted from the RGB video easily and efficiently. To improve the recognition accuracy and reduce the computational cost, this paper proposes a multi-scale GCN with data preprocessing and attention modules to extract spatiotemporal information and combine multi-stream features for skeleton-based violent action recognition. Firstly, the spatial GCN with the attention module is constructed to extract the multi-scale spatial features by learning the adjacency information between the multi-order joints and build the channel-based dependencies with a low number of parameters. And then, a temporal GCN in the form of hybrid dilation convolution to obtain different sizes of perceptual fields and extract the multiscale contextual information by setting different dilation convolution rates. Finally, the accuracy of recognition is further improved by fusing the multi-stream features related to human joints and bones.

The main contributions of this paper are as follows:

(1) In the spatial space, we design a multi-scale spatial GCN with a fused channel attention mechanism to extract spatial information and the correlation features between channels.
(2) In the temporal space, we propose a temporal convolution network in the form of hybrid dilation convolution to extract the temporal features from skeleton sequences, which can be used to capture multi-scale contextual information and reduce the number of network parameters.
(3) The model incorporates joint position, joint motion, bone vector and bone motion information to further improve the accuracy of violent action recognition.

## 2. Related works

In the field of computer vision, deep learning approaches have become the dominant research direction in tasks such as image classification and target detection since they have a better ability to capture distinguishing features. In this paper, three categories of deep learning methods based on skeleton sequences are briefly reviewed: CNN, RNN, and GCN.

### 2.1. CNN-based methods

Since CNNs can learn high-level semantic information efficiently and effectively, they are usually widely used in image processing tasks. However, it is difficult and challenging to balance and make full use of spatiotemporal information for human action recognition based on skeleton sequences (Kim and Reiter, 2017). The mainstream approaches usually represent skeleton sequences as pseudo images as the standard input of CNNs (Cao et al., 2018; Hou et al., 2018; Xu et al., 2018; Li C. et al., 2019). In these methods, the spatial structure and temporal dynamic information of the skeleton sequences are encoded as columns and rows of a tensor, respectively. Caetano et al. (2019b) proposed a method to represent skeletal motion information based on convolution neural networks, which first encoded the temporal dynamic information by calculating the magnitude and direction values of the joint motion, and then different time scales were used to filter the noisy motion information for capturing long-distance joint point dependence. In addition, Caetano et al. (2019a) introduced reference nodes and tree structures to represent the skeleton image through the framework of the SkeleMotion method, the former incorporating different spatial information among the articulations, but the latter preserving important spatial relationships by traversing a skeleton tree with a depth-first algorithm. By considering only adjacent joints within the convolution kernel to learn co-occurring features, some potentially associated joints are ignored. Therefore, Li C. et al. (2018) used an end-to-end network framework to learn co-occurrence features by a hierarchical approach in which contextual information is gradually aggregated at different layers. First, point-level information is encoded independently for each node. Then, combining them into semantic representations in the temporal and spatial domains, respectively.

### 2.2. RNN-based methods

The RNN-based approaches essentially uses the output of the previous frame as the input of the current frame, which allows continuous sequential data to be processed efficiently. To remedy the gradient disappearance and long-range modeling problems of standard RNN, researchers have proposed improved RNNs such as long short-term memory neural network (LSTM) and gated neural unit (GRU), which model the spatiotemporal dimension to capture the correlation features between sequence data (Liu et al., 2018; Song et al., 2018; Jiang et al., 2020; Shu et al., 2021). Wang and Wang (2017) proposed a two-stream recurrent neural network to model spatiotemporal information by using 3D transforms-based data enhancement techniques. To extract more distinguished spatiotemporal features, Song et al. (2017) proposed two spatiotemporal attention sub-modules based on LSTM networks and designed a spatial attention sub-module based on the joint selection gate, which can adaptively assign attention weights to the skeleton nodes in each frame. Meanwhile, the temporal attention sub-module based on the frame selection gate is designed to assign different attention weights to different frames for the extraction of keyframes. A longer and deeper RNN network is proposed by Li S. et al. (2018) to solve the gradient explosion and disappearance problem, which be constructed to learn high-level semantic features with better robustness. Furthermore, due to the strong capability of CNNs for spatial feature extraction, Li C. et al. (2022) combined RNN and CNN models to improves the spatiotemporal modeling capability in complex scenes, as RNN is mainly used for temporal modeling and CNN is mainly used for spatial modeling.

### 2.3. GCN-based methods

The human skeleton sequence is inherently a topological graph, rather than a Euclidean spatial image based on CNNs or a segment of sequence vectors based on RNNs methods. The spatiotemporal dependencies between the associated vertices cannot be fully expressed by simply transforming the sequence into a two-dimensional pseudo-image or sequence vector. The GCN is developed based on CNN (Gao et al., 2019; Si et al., 2019; Wu et al., 2019; Degardin et al., 2021; Tu et al., 2022), which can be used to efficiently capture spatial features information by adjusting the convolution kernel size with different neighbors of each vertex. Yan et al. (2018) proposed a spatiotemporal graph convolutional neural network (ST-GCN) for human behavior recognition, which consider human joints as vertices of a graph and connections between joints and different frames of the same joints as edges of the graph. By designing different convolutional kernel strategies for modeling, the spatiotemporal features between joints are captured and the action is predicted by a Softmax classifier. As the skeleton graph used in ST-GCN, there is an implicit problem of missing node-dependence. To obtain richer inter-joint dependencies, Li M. et al. (2019) proposed an action-structural graph convolutional neural network (AS-GCN) with an actional-links module to extend the skeleton graph to represent higher-order dependencies and capture the potential dependencies of a specific action. Shi et al. (2019b) proposed a two-stream adaptive graph convolution network (2s-AGCN) for adaptive learning of spatiotemporal features from skeleton sequences in end-to-end networks. Similarly, Li B. et al. (2019) proposed a spatiotemporal graph routing (ST-GR) approach to capture the intrinsic higher-order connectivity relationships among the skeleton joints, which added additional edges to the network skeleton graph through a global self-attentive mechanism. Liu Z. et al. (2020) proposed a decomposed multiscale aggregation method and a spatiotemporal graph convolution operator (G3D) to implement a powerful feature extractor. Zhang et al. (2020) proposed a simple effective semantics-guided neural network (SGN) to obtain higher-order semantic information of the nodes for skeleton-based action recognition. To reduce the computational cost of the GCN, Cheng et al. (2020) designed a Shift-GCN that employs a shift-graph operation and a point-level convolution form instead of using standard graph convolution. Along this line of research, Song et al. (2022) proposed a multi-stream GCN model that incorporates input branches including joint position, motion velocity and skeletal features at an early stage, and utilizes separable convolutional layers and a composite scaling strategy to reduce significantly redundant trainable parameters while increasing model capacity. Recently, Chen et al. (2021) proposed a channel-level topology refinement graph convolution (CTR-GC) based on dynamic topology and multi-channel feature modeling. Specifically, CTR-GC takes the shared topology matrix as the entire prior for a channel and then refines it by inferring channel-specific correlations to obtain a channel-level topology. Li et al. (2021) proposed an Elastic Semantic Network (Else-Net), which consists of a GCN backbone model and multiple layers of elastic units for continuous human behavior recognition. In particular, each flexible unit contains several learning blocks to learn diverse knowledge from different human behaviors, with a switch block to select the most relevant block for the newly entered behavior. Chi et al. (2022) proposed InfoGCN that includes an information bottleneck goal to learn maximally informative action representations and an attention-based graph convolution to infer contextually relevant skeletal topology.

## 3. Proposed method

### 3.1. Overall framework

**Figure 1:**
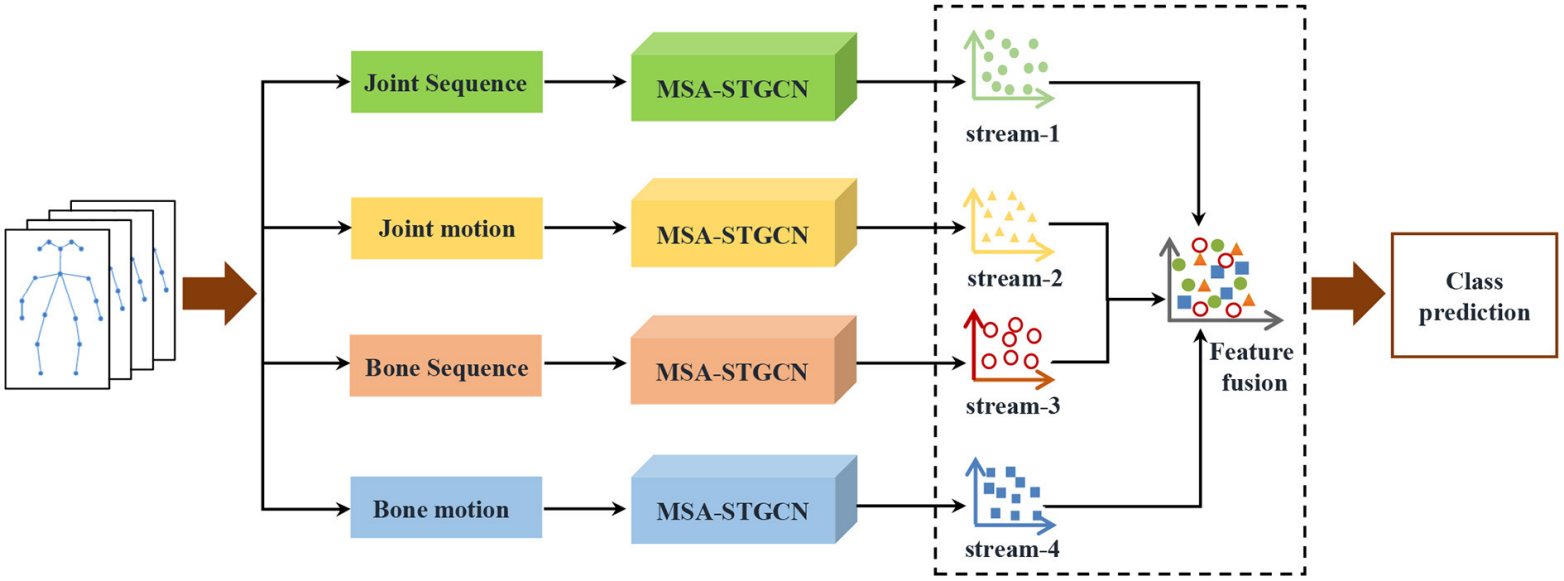
Multi-stream fusion violence action recognition framework.

Inspired by the success of the two-stream framework and graph convolution (Shi et al., 2019b, 2020), this paper proposes a multi-scale attention spatiotemporal graph convolution network (MSA-STGCN) to recognize violence human actions from different perspectives, as shown in Figure 1. First, the original joint data are preprocessed to obtain joint position, bone vector, joint motion and bone motion information. Then, these four categories of data are input into our designed MSA-STGCN, respectively. Finally, the four-stream features are fused using a weighted summation method to predict the action category.

### 3.2. The proposed MSA-STGCN

**Figure 2:**
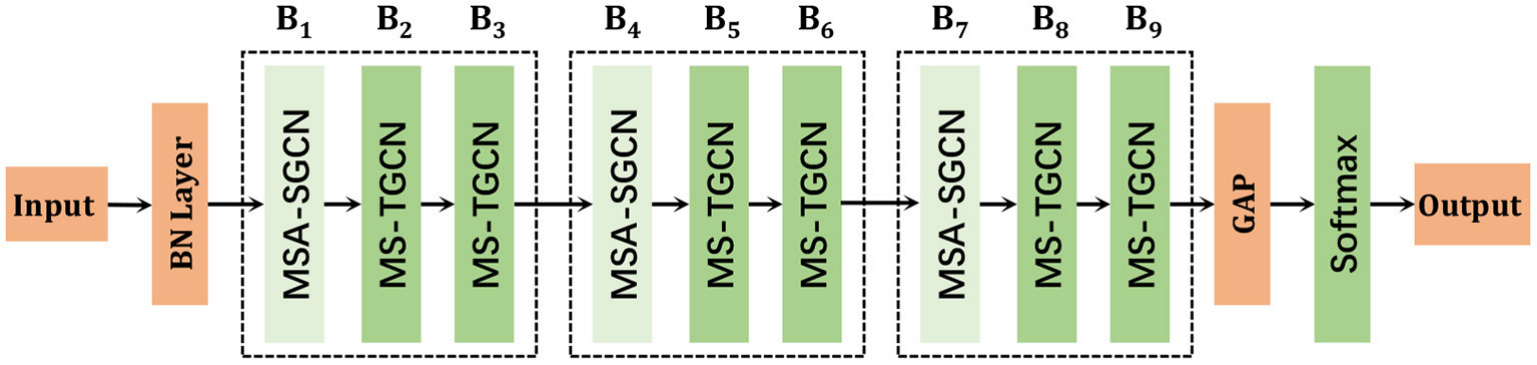
Multi-scale attention spatiotemporal graph convolution network.

The proposed MSA-STGCN consists of nine spatiotemporal feature extraction modules, as shown in Figure 2. Given a skeleton sequence *X* ∈ ℝ^*C*×*T*×*V*^, where *C*, *T*, and *V* are the number of channels, sequences and joint points of the input data, respectively. Among them, the batch normalization layer (BN) normalizes the input data *X*, the output feature size of modules B_1_ to B_3_, B_4_ to B_6_, and B_7_ to B_9_ are *B*×*C*×*T*×*V*, *B*×*C*×*T*/2 × *V*, and *B*×*C*×*T*/4 × *V*, respectively, where *B* is the number of batch size, and the number of output channels of modules are 96, 96, 96, 192, 192, 192, 384, 384, and 384, respectively. Modules B_1_, B_4_, and B_7_ adopt the multi-scale attention enhanced spatial graph convolution network (MSA-SGCN) to extract the spatial features, while modules B_2_, B_3_, B_5_, B_6_, B_8_, and B_9_ use multi-scale temporal graph convolution networks (MS-TGCN) to obtain the temporal feature from skeleton sequences. Then, global average pooling (GAP) layer is applied to aggregate the spatiotemporal features and unify the feature graph size of the samples. Finally, the Softmax layer is used to obtain the classification probability and prediction category.

#### 3.2.1. Multi-scale attention enhanced spatial graph convolution network

The effectiveness of the attention mechanism has been demonstrated in tasks such as target detection and image classification, which has been gradually introduced into the field of action recognition. In this paper, we design a channel attention module based on the Squeeze-and-Excitation Networks (SE-Net) (Hu et al., 2020), named multi-scale attention Spatial Graph Convolution Network (MSA-SGCN), to automatically learn the correlation and significance between feature map channels. The SE-Net improves the feature description capability by modeling the dependencies of each channel, which enhances useful features and suppress non-useful features by adaptively adjusting the feature response values of each channel. Motivated by these advantages, we insert the Squeeze-and-Excitation module to a spatial graph of the convolution neural network to obtain more contextual feature through automatically learning the importance of different channel features. The earliest application of GCNs to human action recognition tasks is ST-GCN, where spatiotemporal graph convolution and spatial division strategies are used to model skeleton sequences to extract feature information in the spatial space (Yan et al., 2018). In contrast, a multi-scale spatial and motion graph convolution modules are designed in STI-GCN (Huang et al., 2020) to extract and merge features for topological graphs from multiple perspectives.

**Figure 3:**
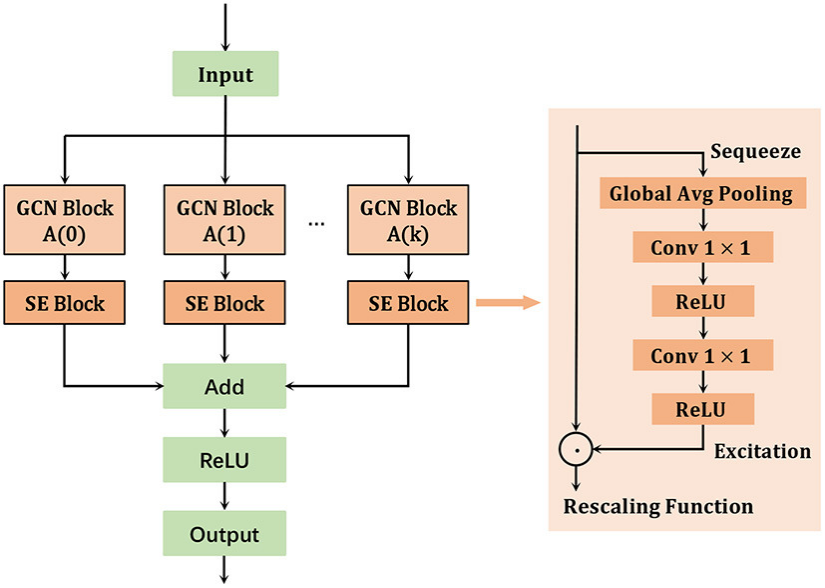
Multi-scale attention enhanced spatial graph convolution network.

Based on the success of these models, we design a multi-scale attention spatial graph convolution network to learn spatial features from skeleton sequences, as shown in Figure 3. The feature extraction for each input layer is performed by


(1) $$\begin{array}{l} X_{{\;}_{t}}^{l+1}=ReLU\left(\underset{k}{\sum }D_{k}^{-\frac{1}{2}}A_{k}D_{k}^{-\frac{1}{2}}X_{t}^{l}W_{k}^{l}\right) \end{array}$$

where *k* controls the scale size of the whole network and also represents the shortest distance between the nodes *V*_*i*_ and *V*_*j*_. *A*_*k*_ represents the relationship matrix between the current node and the *k*-hop neighbors, which includes the self-loop connections. It allows the model to learn information about the neighbor's features between each node. *D*_*k*_ denotes the square root of the inverse of the degree matrix of the neighborhood matrix *A*_*k*_, which is used for symmetric normalization of the neighborhood matrix *A*_*k*_. In the calculation, the features of the node itself have been calculated as well as the weighted sum of the features of all neighbors. *X*_*t*_ represents the input of the frame and denotes the number of layers of the network. *W*_*k*_ is the current node, *W*_*k*_ is a learnable weight matrix between the *k*-hop neighbors of the current node, which implements the edge importance weighting. *Relu*() represents the activation function.

In the proposed MSA-SGCN, the scale of each model is adjusted by *k* to obtain different scale feature information in the spatial space, and the multi-order neighborhood information is aggregated to obtain all the neighborhood feature information of each joint. In addition, attention operations are performed on each scale output feature in the channel dimension to automatically learn the correlation contextual information between feature map channels.

#### 3.2.2. Multi-scale temporal graph convolution network

Existing methods usually use standard convolution with fixed kernel size throughout the network module to model the temporal information (Yan et al., 2018; Li M. et al., 2019; Shi et al., 2019a,b). In this paper, we proposed a multiscale aggregation learning method by introducing hybrid dilation convolution to improve the traditional temporal convolution module (Ople et al., 2020). Because of the exponential expansion of the perceptual field with guaranteed coverage, the proposed MS-TGCN can effectively aggregate multi-scale contextual information without loss of resolution by using dilation convolution. However, the result of a certain layer of null convolution is not dependent on the information of the previous layer due to the grid effect problem of the dilation convolution, and the information obtained from the long-distance convolution lacks relevance. Therefore, this model avoids the grid effect problem by introducing a hybrid form of dilation convolution (Wang et al., 2018). At the same time, the model takes the feature map *X* as input without introducing additional parameters and generates a feature map of the same size with the same dimension, which is passed to the next network module.

**Figure 4:**
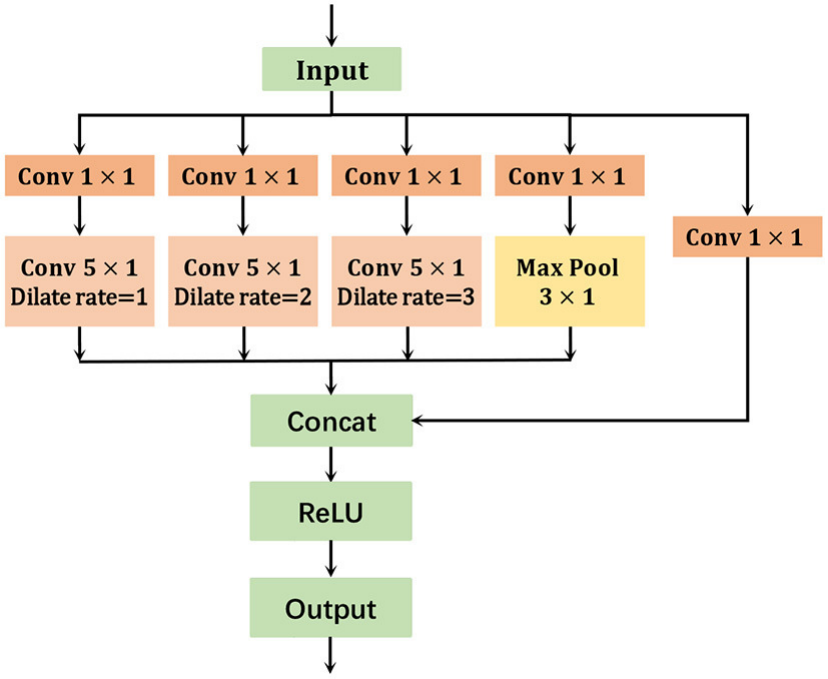
Multi-scale temporal graph convolution network.

As shown in Figure 4, the number of model parameters is reduced by adopting a multi-branch structure and passing each branch through a convolution kernel of size 1 × 1. The size of the convolution kernels in each branch is modified to 5 × 1, which gives a larger perceptual field than the convolution kernel size of 3 × 1. In addition, we also set the convolution rate of different sizes of holes, 1, 2, and 3 to obtain different scales of the same feature map for avoiding the problem of gradient disappearance. Finally, the aggregation layer fuses the multi-scale information and passes it to the next module of the network. The proposed model can learn richer temporal features and reduce the number of parameters after replacing the regular temporal convolution method.

### 3.3. Representation of skeleton sequences

The position of the joint points of the human skeleton is defined as:


(2) $$\begin{array}{l} V_{i,t}=\left(x_{i,t},y_{i,t},z_{i,t}\right),\forall i\in N,t\in T \end{array}$$

where *N* is the number of joints in the human skeleton, *T* is the total number of sequences, and *i* represents the joints at time *t*. In 3D skeleton sequences, the joint positions consist of three position coordinates (*x, y, z*), which are usually captured directly by a depth camera or extracted from RGB video data.


(3) $$\begin{array}{l} B_{i,j,t}=V_{j,t}-V_{i,t}=\left(x_{j,t}-x_{i,t},y_{j,t}-y_{i,t},z_{j,t}-z_{i,t}\right) \end{array}$$

In particular, the joint near the center of gravity of the human skeleton is defined as the source node with coordinates denoted as *V*_*i, t*_, while the joint far from the center of gravity is defined as the target node with coordinates denoted as *V*_*j, t*_. Since each joint has no self-loop, each bone can be assigned a unique joint point, forming a directed acyclic graph. In addition, since the root node does not have any bones assigned to it, to simplify the network design, the vector of bones assigned to the root node is set to 0.

The definition of human joint motion information is defined as:


(4) $$\begin{array}{l} J\text{-}M_{i,t+1}=V_{i,t+1}-V_{i,t}=\left(x_{i,t+1}-x_{i,t},y_{i,t+1}-y_{i,t},z_{i,t+1}-z_{i,t}\right) \end{array}$$

where *V*_*i, t*_ represents the position coordinates of the *i*^*th*^ joint at time *t*:(*x*_*i, t*_, *y*_*i, t*_, *z*_*i, t*_), and *V*_*i, t*+1_ represents the position coordinates of the *i*^*th*^ joint at time *t*+1:(*x*_*i, t*+1_, *y*_*i, t*+1_, *z*_*i, t*+1_), and the position of the same joint in adjacent frames are difference to obtain the sequence of joint motion information.

The definition of human bone motion information is defined as:


(5) $$\begin{array}{l} B\text{-}M_{i,j,t,t\text{+ 1}}=B_{i,j,t+1}-B_{i,j,t} \end{array}$$

where *B*_*i, j, t*_ represents the skeletal vector information at time *t*, and *B*_*i, j, t*+1_ represents the skeletal vector information at time *t*+1. We capture the skeletal motion information by the difference of adjacent skeletal vectors. The fusion strategy is used to gather the features of nodal position information, skeletal vector information, nodal motion information, and skeleton motion information streams.

### 3.4. Implementation details

The configuration information of the experimental platform is Intel Xeon Silver 4210R CPU with 2.40GHz, 80G memory, 1TB SSD storage, and RTX3090. The number of samples per training batch (Batch size) is set to 32, and the cross-entropy function is used as the loss function for gradient back propagation. The number of iterations (Epoch) is set to 80, and the weight decay parameter is set to 0.0005.The initial learning rate is set to 0.05, and the learning rate is adjusted at a given interval by dividing the learning rate by 10 when the 30*th* Epoch and 40*th* Epoch are reached, respectively.

## 4. Experiments

### 4.1. Datasets

In this paper, we conducted experiments on two datasets: Filtered NTU RGB+D and Kinetics Skeleton 400. The Filtered NTU RGB+D dataset is based on the NTU RGB+D 120 dataset (Liu J. et al., 2020) by discarding other daily movements and filtering out 10 types of movements to form a skeleton dataset. The Kinetics Skeleton 400 dataset is based on the Kinetics-400 dataset (Carreira and Zisserman, 2017) by preprocessing each frame of the original RGB video with a pose estimation algorithm to extract the skeleton sequence data to form a 400 classes normal motion dataset.

#### 4.1.1. Filtered NTU RGB+D

**Figure 5:**
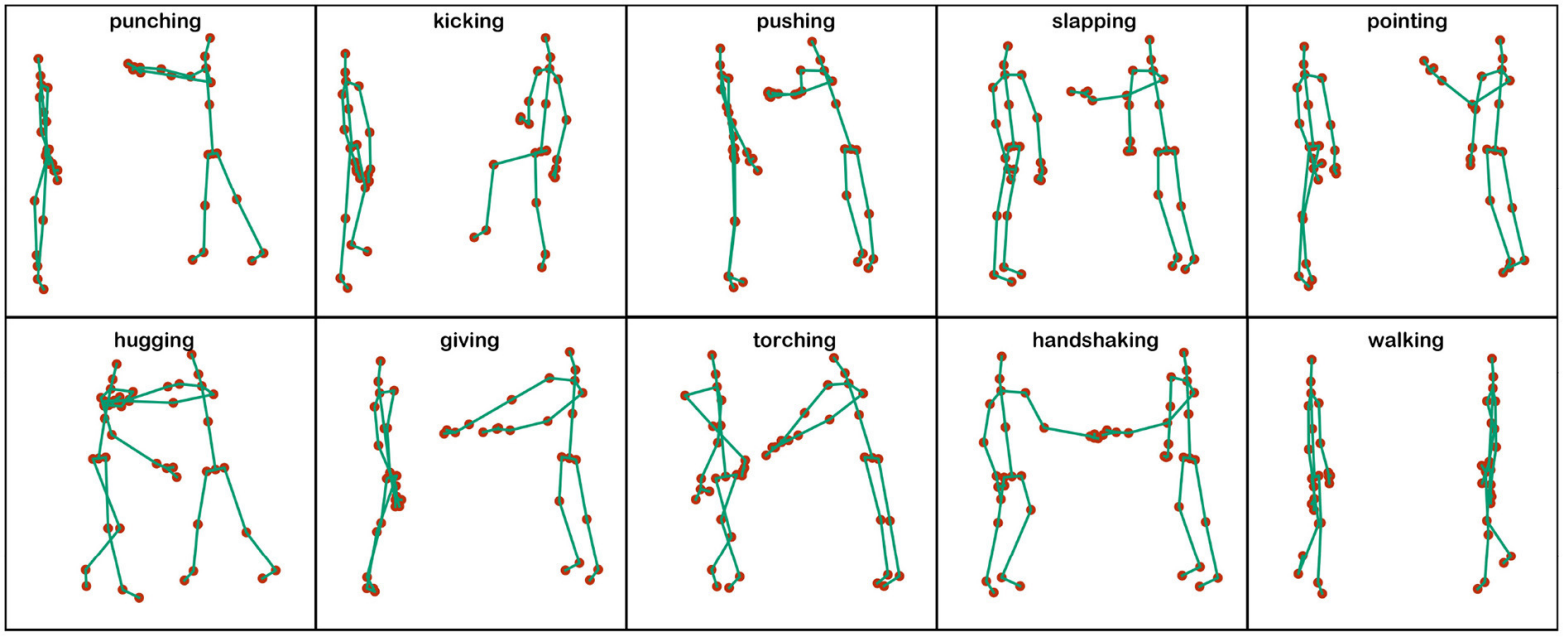
Visual representation of 10 types of human violence actions.

The NTU RGB+D 120 is the largest and most widely used indoor motion dataset, containing 114,400 motion clips in 120 categories. Each clip was performed by 40 volunteers ranging in age from 10 to 35 years old, and each action was filmed from different angles using three Kinect V2 cameras. The previous violence dataset is mainly RGB, depth information, and optical flow modality, while NTU RGB+D 120 is 3D skeleton data, which contains 3-dimensional coordinates of 25 body joints in each frame. Meanwhile, to compare the traditional graphical neural network in a violence recognition task, this paper takes 120 classes of NTU RGB+D 120 dataset for filtering, and finally selected 10 classes of skeleton data about human interaction actions, and the final action types are visualized as shown in Figure 5, including walking, pushing, punching, pointing, slapping, shaking hands, touching, hugging, giving and kicking, among which pushing, punching, kicking, pointing and slapping are the five kinds of video the common violent actions in surveillance. In this paper, we mainly study the recognition of violent actions in surveillance video, and the application scenario is usually the recognition of actions from a certain viewpoint for different objects. Therefore, we adopt a Cross-subject (X-Sub) protocol from the recommended benchmark of the original paper and reports the Top-1 accuracy in the experiment.

#### 4.1.2. Kinetics Skeleton 400

Kinetics-400 is a large human action dataset with 300,000 video clips from the YouTube video site. It covers 400 human action categories from daily life, sports scenes, and complex human interactions. However, this dataset only provides raw RGB video clips without skeleton data. In this work, since the concentration is on skeleton-based action recognition, so we use the OpenPose pose estimation method for preprocessing to extract the coordinates of human joint positions for each frame of each clip. For a multi-person action scene, the two persons with the highest average nodal confidence are selected. In this way, an RGB segment with T-frames is converted into a skeleton sequences. The final dataset consists of a training set of 240,000 segments and a validation set of 20,000 segments. In this paper, we compare the models on the training set and report the accuracy of the validation set. Referring to the evaluation methods proposed in Yan et al. (2018) and Liu Z. et al. (2020), we trains the model on the training set and reports the accuracy of Top-1 and Top-5 on the validation set.

### 4.2. Effectiveness of the proposed method

**Figure 6:**
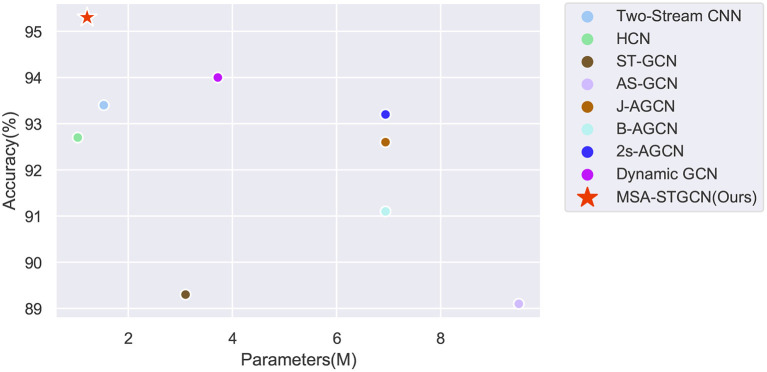
The accuracy and parameters of the proposed method compared to other methods on the Filter NTU RGB+D dataset.

On the Filtered NTU RGB+D dataset, we have done comparison experiments on two CNN-based methods, namely Two-Stream CNN and HCN model, and on four GCN based methods, namely ST-GCN, AS-GCN, 2S-AGCN and Dynamic GCN network, and the results are shown in Figure 6 and Table 1. The major evaluation metrics taken include accuracy and parameters, and the proposed model achieves relatively great results for both in comparison, with an accuracy of 95.3% and parameters of only 1.21M, which reflect the effectiveness and efficiency of the proposed MSA-STGCN. Due to the limited modeling capability of the compared baseline model, it lacks consideration of the spatiotemporal dependencies between skeleton sequences, whereas the proposed model can obtain the long and short temporal dependencies between each frame's articulation points by combining multi-scale and channel attention mechanisms in spatio-temporal modeling. As a result, the proposed model shows a significant improvement in recognition accuracy compared with existing GCNs, and it improves by 2.1% compared with the best 2s-AGCN. Due to the multi-branching structure of the model in both temporal and spatial dimensions, and the eventual aggregation of multi-scale information, the number of parameters of the proposed model is substantially reduced. This effectively validates the accuracy and computational cost advantages of the model proposed for violent action recognition tasks.

**Table 1:** Comparison of different algorithms on Filtered NTU RGB+D dataset.

**Methods**	**Accuracy (%)**	**Params (M)**
Two-Stream CNN	93.4	1.53
HCN	92.7	1.03
ST-GCN	89.3	3.10
AS-GCN	89.1	9.50
J-AGCN	92.6	6.94
B-AGCN	91.1	6.94
2s-AGCN	93.2	6.94
Dynamic GCN	94.0	3.72
**Ours**	**95.3**	**1.21**

The bold values indicate the results of our proposed method (MSA-STGCN).

The main indicators of evaluation include accuracy and the number of parameters. The compared baseline models have limited modeling capability and lack the consideration of spatiotemporal dependencies among skeleton sequences, while the proposed model can obtain the long-term dependencies of an active state by combining multi-scale and channel attention mechanisms in the spatiotemporal modeling. Therefore, the proposed model has a significant improvement in recognition accuracy compared with the baseline model, which has improved by 2.1% compared with the best 2s-AGCN (Shi et al., 2019b). The proposed multi-information flow fusion method could fully exploit the specific relationships of the original data to further improves the recognition performance. The number of parameters of the proposed model can be reduced to 1.21M due to the multi-branch structure of the model in time and space dimensions, which effectively validates the accuracy and computational cost advantages.

**Figure 7:**
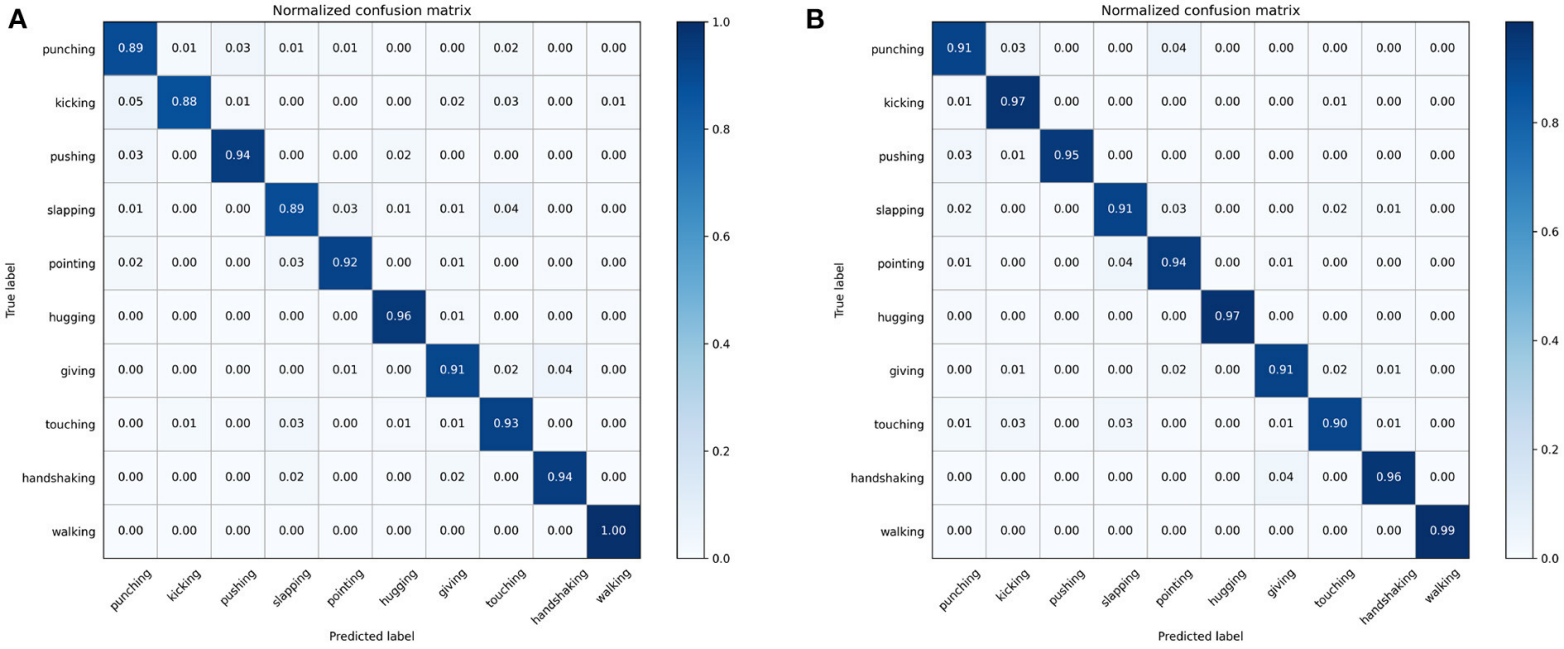
Normalized confusion matrix of 10 types of human action. **(A)** Confusion matrix of 2s-AGCN model. **(B)** Confusion matrix of our model.

Meanwhile, the 10 types of actions on the Filtered NTU RGB+D dataset: punching, kicking, pushing, slapping, pointing, hugging, giving, touching, handshaking, and walking were recognized, and the results are shown in Table 2. The recognition accuracy of these 10 types of actions were 91.1, 96.5, 94.7, 91.0, 93.6, 96.8, 91.5, 90.2, 96.0, and 98.6%, respectively. Normalized confusion matrix of 10 types of human action as shown in Figure 7, which illustrates that the method can be applied to violence recognition tasks in practical applications.

**Table 2:** Comparison of recognition results for 10 types of human action on the Filtered NTU RGB+D dataset.

**Classes**	**Samples**	**True**	**Accuracy (%)**	
Punching	271	247	91.1	
Kicking	260	251	96.5	
Pushing	281	266	94.7	
Slapping	278	253	91.0	
Pointing	266	249	93.6	
Hugging	278	269	96.8	
Giving	281	257	91.5	
Touching	287	259	90.2	
Handshaking	273	262	96.0	
Walking	277	273	98.6	

To further validate the generalization capability of the proposed recognition framework, we further conduct experiment on the Kinetics Skeleton 400 dataset, and Table 3 shown the results of the comparison experiments with ST-GCN, AS-GCN, ST-GR and 2s-AGCN. It can be seen that the proposed model achieves 36.2 and 58.5% accuracy in Top-1 and Top-5, respectively, which are still significant improvements compared to some of the baseline models. The results demonstrate that the proposed model can capture more features by combining multi-scale attention mechanisms, which can effectively identify more details in multi-frame skeleton sequences.

**Table 3:** Comparison of different algorithms on Kinetics Skeleton 400 dataset.

**Methods**	**Top-1(%)**	**Top-5(%)**
ST-GCN	30.7	52.8
AS-GCN	34.8	56.5
ST-GR	33.6	56.1
2s-AGCN	36.1	**58.7**
**Ours**	**36.2**	58.5

The bold values indicate the best accuracy.

### 4.3. Ablation study and discussion

#### 4.3.1. Attention mechanism

This part mainly verifies the effectiveness of the attention mechanism proposed in the recognition framework by inserting the attention mechanism in the spatial dimensional to graph convolution network (ASGCN), and the experimental results are shown in Table 4. Firstly, the input skeleton sequences were tested for joints and bones in the spatial graph convolution layer (SGCN) without the SE Block, which was represented by J-ASGCN w/o SE and B-ASGCN w/o SE, respectively. Then, the results of the two data streams are fused and represented by ASGCN w/o SE. Finally, the SE Block attention mechanism is introduced in SGCN, and the model with the nodal position as input is represented by J-ASGCN, and the model with the skeletal vector as input is represented by B-ASGCN.

**Table 4:** Comparison of spatial graph convolution layer with and without SE block on the Filtered NTU RGB+D dataset.

**Methods**	**Accuracy (%)**
J-ASGCN w/o SE	93.6
B-ASGCN w/o SE	93.1
ASGCN w/o SE	94.3
J-ASGCN	94.0
B-ASGCN	93.2
**ASGCN**	**94.9**

The bold values indicate the accuracy of the model incorporating the attention mechanism.

**Figure 8:**
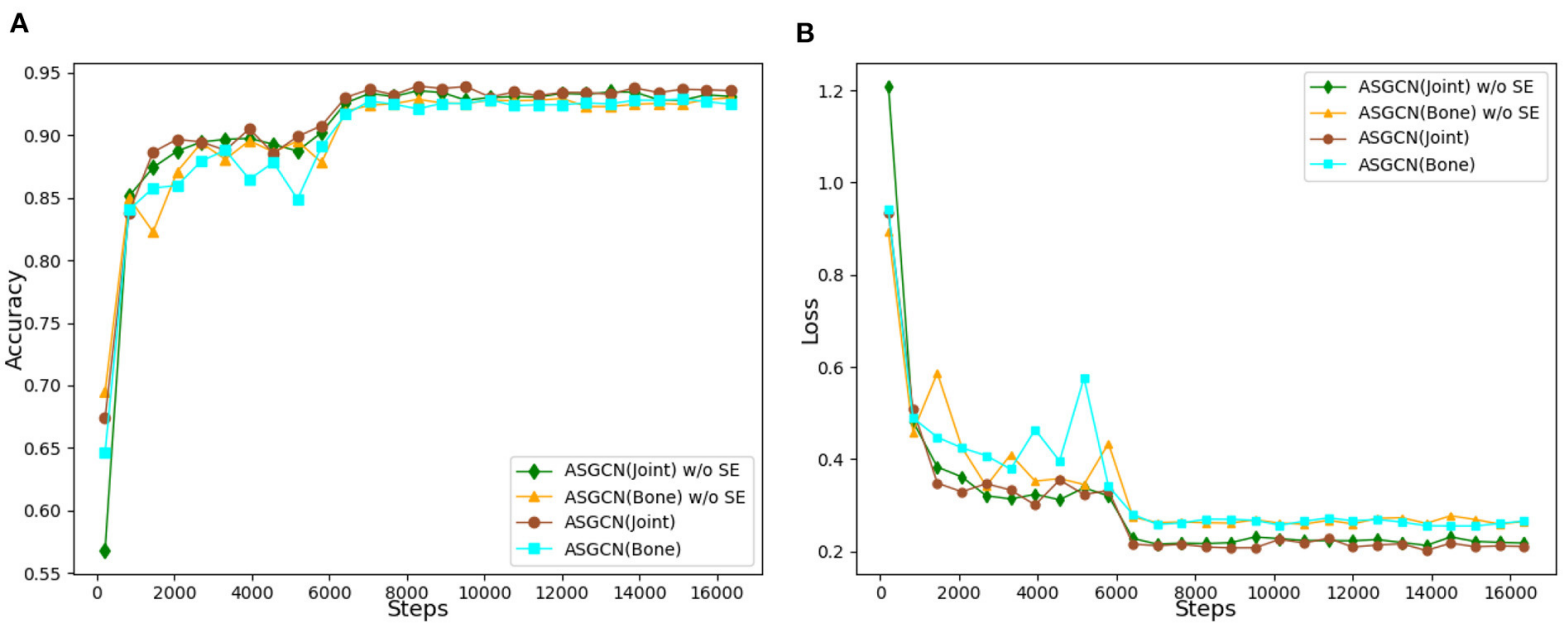
**(A)** Accuracy comparison of spatial graph convolution layer with or without SE block. **(B)** Loss function comparison of spatial graph convolution layer with or without SE block.

The variation accuracy of networks and the loss function values during the whole training process is shown in Figure 8. The recognition accuracy of J-ASGCN obtain 94.0% in the joint position information stream (increase of 0.4%), the B-ASGCN achieve 93.2% (increase of 0.1%) in the bone vector information stream, and the ASGCN achieved 94.9% (increase of 0.6%). Throughout the training process of the model, the accuracy of the test was improved rapidly in the early stage of the experiment, reaching about 85%, which is due to the high optimization efficiency of the proposed multi-scale spatial graph convolution. As the number of iterations increases, the final test accuracy and loss function converge very well, and the test accuracy and loss function curves are smoother in the later stage. Therefore, the attention mechanism SE Block does not play a significant role in this layer since the spatial feature extraction performance of the spatial map convolution layer itself is very robust. However, adding SE Block to our model can optimize the learning content and obtain more useful feature information, thus verifying the effectiveness of the method.

#### 4.3.2. Hybrid dilation convolution

Without pooling loss, the dilation convolution can increase the perceptual field of the feature map so that the output of each convolution contains a larger range of feature information. In this paper, we consider obtaining different sizes of perceptual fields in the temporal dimension to achieve a multi-scale fusion training network. To verify this idea, firstly, we compare the convolution rates of different sizes of voids, which are set to 1, 2, and 3, and the corresponding accuracy rates are 93.1, 93.2, and 93.5 respectively, as shown in Table 5. It is obvious that the accuracy of the model recognition is in a stable state with the increase of the hole convolution rate, which is not a very good training effect. Considering that the increase in the convolution rate of the dilation will bring about a grid effect, which will lead to the loss of continuity of a certain part of the feature information, and even, probably, the important feature information as well. Therefore, this paper solves the problem of discontinuity in the convolution kernel by designing a hybrid dilation convolution (HDC) form of temporal map convolution network, represented by MS-TCN(HDC). Finally, the accuracy of the MS-TCN(HDC) model reached 94.0% by fusing the hybrid dilation convolution form with different dilation rates.

**Table 5:** Accuracy comparison of different dilated convolution rates used in temporal graph convolution layer on the Filtered NTU RGB+D dataset.

**Methods**	**Accuracy (%)**
MS-TCN(dilate rate = 1)	93.1
MS-TCN(dilate rate = 2)	93.2
MS-TCN(dilate rate = 3)	93.5
**MS-TCN(HDC)**	**94.0**

The bold values indicate the accuracy of the model using hybrid ablation convolution.

**Figure 9:**
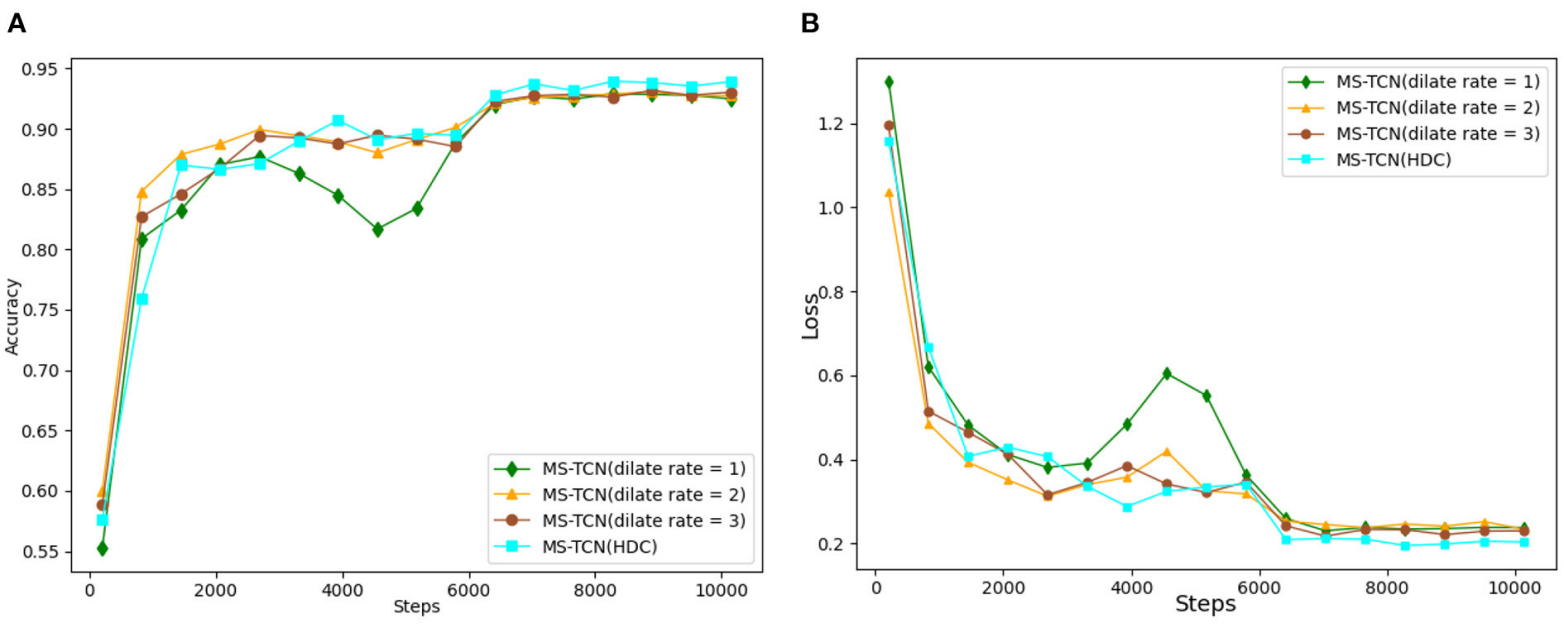
**(A)** Comparison of recognition accuracy with different dilated convolution rates. **(B)** Comparison of loss function with different dilated convolution rates.

The variation in the test accuracy of each network and the variation loss throughout the training process is shown in Figure 9. In the early stage of the experiment, the speed of convergence of the loss function increased slightly with the increase of the hole convolution rate, and the speed of test accuracy also increased. By adjusting the dilation convolution rate, the scale of the model is increased and the parameters of the network are changed, thus slightly improving the optimization efficiency of the network in the early stage of training. As the number of iterations increases, the final validation accuracy increases with the increase of the dilation convolution rate, and the training loss function achieves good convergence and a smoother curve in the later stages of training. The experimental results verify that the graph convolution network model constructed in the form of hybrid dilation convolution can learn more time-domain feature information at multiple scales compared with single dilation convolution.

#### 4.3.3. Multi-stream fusion

Finally, the proposed multi-stream model incorporating joint position information, bone vector information, joint motion information, and bone motion information was tested and the experimental results are shown in Table 6. As for the input models of node position information, bone vector information, node motion information, and bone motion information, the corresponding accuracy rates were 94.0, 93.2, 92.1, and 93.4% for J-MSAGCN, B-MSAGCN, J-M-MSAGCN, and B-M-MSAGCN, respectively. The accuracy of MS-AGCN with a multi-stream fusion model could reach 95.3%.

**Table 6:** Accuracy comparison of different data stream recognition on the Filtered NTU RGB+D dataset.

**Models**	**Accuracy (%)**
J-MSAGCN	94.0
B-MSAGCN	93.2
J-M-MSAGCN	92.1
B-M-MSAGCN	93.4
**MS-AGCN(fusion)**	**95.3**

The bold values indicate the accuracy using multi-stream fusion.

**Figure 10:**
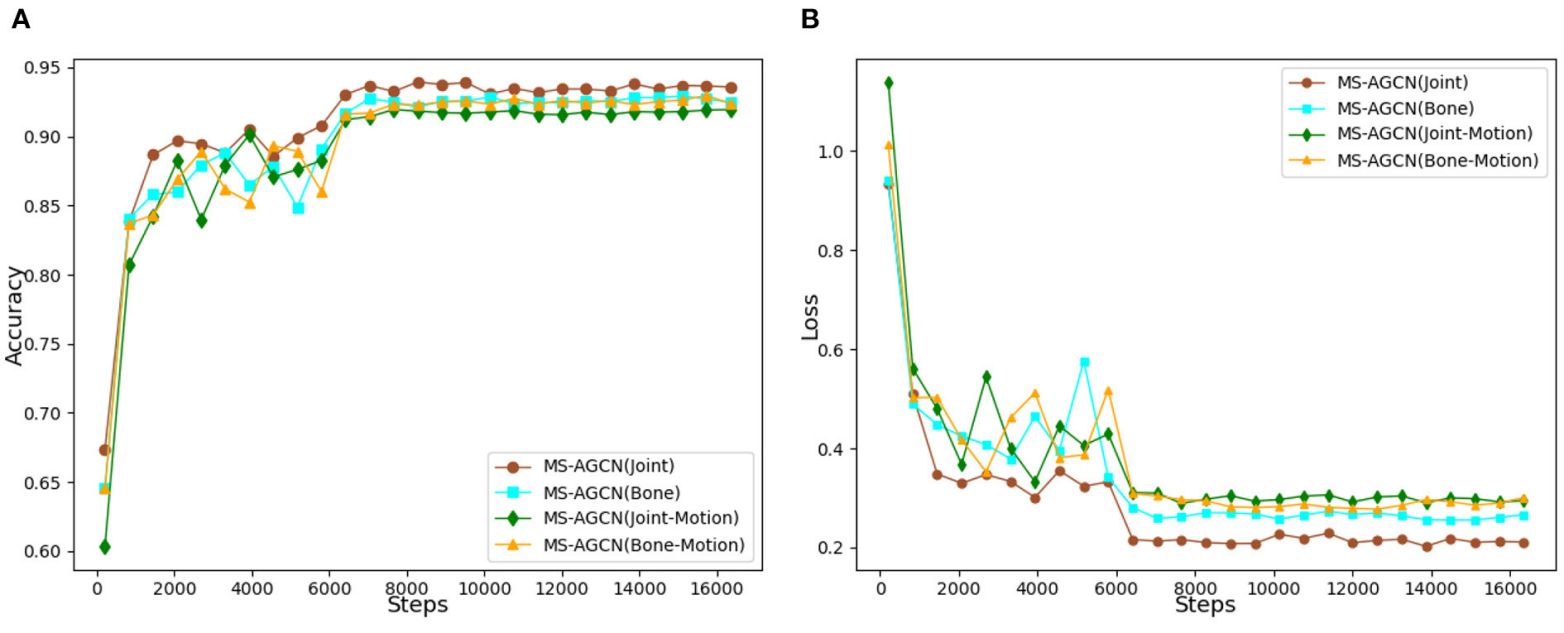
**(A)** Comparison of recognition accuracy of different data streams. **(B)** Comparison of loss functions of different data streams.

During the whole training process, the variation in the accuracy of each network and the variation loss are shown in Figure 10. As the number of experimental iterations increased, the accuracy of the original joint position information stream increased slightly faster than the other three data streams in the early stage of the experiment, and the loss function also converged faster. This indicates that the original joint position plays an important role in characterizing the movement state, while the accuracy of the other streams is increased by 1.3%, which suggests that by calculating the bone vector information, joint point motion information, and bone motion information, a higher weight is given to the more variable streams, thus enhancing the overall model's characterization of the movement. The experimental results show that the accuracy of the multi-stream fusion method is significantly higher than that of the single-stream method. In particular, the accuracy of the multi-stream fusion method has improved relative to the performance of the joint point information stream method. This shows that the skeleton sequence data can be extracted from different angles and the final fusion output can be used to fully characterize the action features.

## 5. Conclusion

In this paper, we design a novel spatiotemporal graph convolution network with attention mechanism to combine multi-stream skeleton features for human violence recognition. The proposed MSA-STGCN utilizes MSA-SGCN and MS-TGCN to learn spatial and temporal information from four types of skeleton data, respectively, and then a average features fusion mechnism is used to implement violence action classification. Compared with other traditional GCNs, the proposed MSA-STGCN achieves 95.3% accuracy on the Filtered NTU RGB+D dataset with only 1.21M model parameters, and the accuracy of Top-1 and Top-5 reached 36.2 and 58.5% on the Kinetics Skeleton 400 dataset, respectively. The experimental results demonstrate that the effectiveness of MSA-SGCN and MS-TGCN in the proposed MSA-STGCN recognition framework. Compared with the other state-of-the-arts, our framework consistently improves the recognition performance on two large skeleton datasets. In the future, more effective fusion and combining strategies that can help to obtain more distinctive complementary features from multimodal data such as RGB and depth sequences. Another future work is to expand more challenging datasets in order to enhance the generalization capability of the model and design RNN skeleton-based framework to learn the spatiotemporal features to improve recognition performance.

## Data availability statement

The original contributions presented in the study are included in the article/supplementary material, further inquiries can be directed to the corresponding author.

## Author contributions

ZR and QZ: conceptualization. HYa: methodology and validation. ZR and HYu: software and writing—review and editing. QZ: formal analysis and investigation. HYu: resources and funding acquisition. QZ and ZZ: data curation and visualization. HYa and ZR: writing—original draft preparation. ZR: supervision. All authors have read and agreed to the published version of the manuscript.
